# Immunotherapy-Associated Hypothyroidism: Comparison of the Pre-Existing With *De-Novo* Hypothyroidism

**DOI:** 10.3389/fendo.2022.798253

**Published:** 2022-03-11

**Authors:** Megan M. Kristan, David Toro-Tobon, Nnenia Francis, Sameer Desale, Athanasios Bikas, Jacqueline Jonklaas, Rachna M. Goyal

**Affiliations:** ^1^ Division of Endocrinology, University of Maryland Medical Center, Baltimore, MD, United States; ^2^ Division of Endocrinology, Mayo Clinic Rochester, Rochester, MN, United States; ^3^ Division of Endocrinology, University of Pennsylvania Health System, Philadelphia, PA, United States; ^4^ Department of Biostatistics and Bioinformatics, Medstar Health Research Institute, Hyattsville, MD, United States; ^5^ Division of Endocrinology, Brigham and Women’s Hospital, Boston, MA, United States; ^6^ Division of Endocrinology, Georgetown University Medical Center, Washington, DC, United States

**Keywords:** immunotherapy, hypothyroidism, check point inhibitor, thyroiditis, hypophysitis

## Abstract

**Background:**

Immunotherapy has revolutionized the treatment of solid malignancies, but is associated with endocrine-related adverse events. This study aims to dissect the natural course of immunotherapy-induced hypothyroidism and provide guidance regarding diagnosis and management in patients with and without pre-existing hypothyroidism.

**Methods:**

A retrospective analysis was conducted using patients who received immunotherapy between 2010‐2019 within a multicenter hospital system. Participants were separated in three groups—those with pre-existing hypothyroidism, those who developed primary hypothyroidism and those with hypophysitis within a year of their first immunotherapy. Serial effects of immunotherapy on thyroid function tests (TFTs) and levothyroxine dosing were evaluated.

**Results:**

822 patients were screened, with 85 determined to have pre-existing hypothyroidism, 48 *de-novo* primary hypothyroidism and 12 *de-novo* hypophysitis. All groups displayed fluctuations in TFTs around weeks 6‐8 of treatment. In the pre-existing hypothyroidism group, the levothyroxine dose was higher at 54 weeks than at baseline with the difference showing a trend towards statistical significance (p=0.06). The observed mean levothyroxine dose was significantly lower than the mean calculated weight-based dose for all groups. This finding was most clinically significant for the *de-novo* hypophysitis group (mean difference: -58.3 mcg, p<0.0001). The mean 0.9 mcg/kg levothyroxine dose at week 54 for the *de-novo* hypophysitis group was statistically lower than the other groups (p=0.009).

**Conclusion:**

It is reasonable to screen with TFTs every 4 weeks, and space out TFTs surveillance to every 12 weeks after week 20. Our findings suggest a more conservative approach for levothyroxine dosing in those developing *de-novo* hypothyroidism, especially hypophysitis, such as initiating at 0.9-1.2 mcg/kg.

## Introduction

In recent years, the discovery of immunotherapy has transformed the management of several malignant conditions (1, 2). As of today, seven immunotherapy medications (Pembrolizumab, Nivolumab, Ipilimumab, Atezolizumab, Durvalumab, Avelumab and Cemiplimab) have been approved by the FDA for treatment of solid tumors such as non-small cell lung carcinoma (NSCLC), melanoma, breast cancer and renal cell carcinoma, among others (3–9). These medications, commonly known as checkpoint inhibitors (ICIs), consist of monoclonal antibodies against several proteins such as PD-1 (programmed cell death protein-1), PDL-1 (programmed death ligand 1 and 2) and CTLA4 (cytotoxic T lymphocyte-associated antigen-4), all of which diminish the immune response against antigens and act as regulators of the immune system. Moreover, the blockade of these regulators leads to augmentation of T cell activation and proliferation which are responsible for the antitumor response of immunotherapy (10).

With the increasing use of immunotherapy, the identification and reporting of the associated toxicities have become extremely important. Although the exact mechanism that leads to these immune related adverse events (irAEs) has not been fully elucidated, it is believed to be an autoimmune response secondary to the blockade of normal immune regulatory pathways (11, 12). Multiple endocrine irAEs have been identified including hypophysitis, adrenal insufficiency and diabetes, but the most frequently reported is the immune related thyroid toxicity (11, 13–16). The endocrinologists need to be aware of those irAEs, and how to manage them as they are becoming increasingly common (17).

Previously described immune related thyroid toxicities include thyrotoxicosis, hypothyroidism and thyroiditis (11, 13, 18). The most common immune related thyroid toxicity is painless thyroiditis which presents with transient thyrotoxicosis followed by primary hypothyroidism. Thyrotoxicosis, defined as decreased TSH with an elevated Free T4 (FT4) or total T3 (TT3), is transient and typically develops 4 to 8 weeks after ICI initiation. Primary hypothyroidism, defined as high TSH associated with low FT4 or TT3, typically ensues 10 to 20 weeks after ICI initiation (19). More rarely, central hypothyroidism due to adenohypophysis impairment (i.e. hypophysitis) can be seen, characterized by low to mid normal TSH levels and low FT4 (13).

The frequency of these disorders is variable and depends on the specific immune ICIs classes (11). The overall incidence of hypothyroidism has been reported to be 6.6% and it is highest when receiving combination therapy with PD-1 plus CTLA4 inhibitors (15, 16, 18, 20). Patients on anti PD-1 or PDL-1 monotherapy have higher risks of developing hypothyroidism than patients on CTLA4 inhibitors (16, 18). Data on thyroiditis are still limited but the incidence ranges from 1.6% with nivolumab monotherapy up to 4.6% with PD-1 and CTLA4 inhibitors combination therapy (20).

Given the rising frequency of patients with various forms of cancer who are now being treated with ICIs, more data are needed regarding the immune related thyroid toxicities associated with these treatments. Moreover, there are no clear guidelines outlining the screening, surveillance, and management of immune related thyroid toxicities. Thereby, this retrospective review aims to evaluate treatment for immunotherapy-induced hypothyroidism and provide guidance on the management of these adverse events in patients with and without pre-existing hypothyroidism.

## Materials and Methods

### Study Design and Sample

This was a retrospective, multi-center, cohort study conducted within a large hospital system from the Washington D.C. Metropolitan Area. An initial screening of the electronic medical records was conducted through Research Electronic Data Capture (REDCap) (21). A total of 822 patients who had received single or double agent immunotherapy between 2010 and 2019 within the hospital system were identified. From this pool, the patients that had a diagnosis of hypothyroidism or had abnormal thyroid function tests were identified to obtain a final sample of 145 participants. A portion of the patients included in the current study received immunotherapy in the setting of clinical trials. Per protocol, thyroid function tests were checked before initiation of immunotherapy and before each dose of immunotherapy.

### Data Collection

A database was built based on an electronic case information form and manual review of the medical records was conducted for each participant. The following demographic and clinical information were recorded: 1) Age, sex and race; 2) Type of cancer; 3) Date of immune therapy initiation; 4) Check point inhibitor(s) used; 5) TSH prior to initiation of immune therapy; 6) TSH and FT4 levels every 2 weeks for the initial 12 weeks of treatment and every 4 weeks from the weeks 16 to 36 of treatment; 6) Use and dose of levothyroxine prior to the initiation of immune therapy; and 7) Use and dose of levothyroxine every 2 weeks for the initial 12 weeks of treatment, and every 4 weeks from the weeks 16 to 36 of treatment.

In order to avoid misclassification of the immunotherapy-induced hypothyroidism diagnosis, the following measures were taken: a) a manual review of the Oncologist/Endocrinologist notes was performed to ensure cases of non-thyroidal illness were not included; b) patients with Head and Neck malignancies represented 5.5% of the cohort, and a manual review of the medical records was performed to ensure hypothyroidism was ICI-related, and not secondary to external beam radiation therapy; c) in cases of patients on tyrosine kinase inhibitors or other medications that could be considered culprits for development of hypothyroidism, a manual review of the Oncologist/Endocrinologist notes was performed to ensure clear documentation that hypothyroidism was ICI-induced.

### Statistical Analysis

The participants were considered to be clinically euthyroid if they had normal TSH (Normal range: 0.5-4.7 mIU/L) and FT4 (Normal range: 0.8-1.7 ng/dL) levels prior to immunotherapy initiation. The sample was separated into three cohorts—those with hypothyroidism predating the first dose of immunotherapy (On levothyroxine replacement or a documented diagnosis of hypothyroidism prior to receiving ICI), and those who developed primary hypothyroidism (Defined as an elevated TSH and low FT4 levels) and hypophysitis (Defined as low FT4 and TSH levels and at least one more anterior pituitary hormonal deficit: central adrenal insufficiency or central hypogonadism) within one year of the first dose of immunotherapy. For the categorization of every patient, the trend and timing of their thyroid function tests as well as documentation from the Oncologist/Endocrinologist was taken into consideration. Patients followed by Endocrinologists for hypophysitis were asked to have FT4 levels checked in the morning before their Levothyroxine dose, or 4 hours after they had taken their Levothyroxine dose. Continuous variables were summarized using mean, median, standard deviation and inter quartile range, and categorical variables were described with counts and percentages. TSH and FT4 variability was evaluated through a logarithmic transformed regression model and levothyroxine dose variability was evaluated trough a piecewise linear regression with repeated measures where separate lines were fit for time before and after week 20.

### Ethical Considerations

This project was approved by the Institutional Review Board at Georgetown University. Participant’s information, confidentiality, and integrity were respected throughout the duration of the study.

## Results

### Study Characteristics

A total of 822 patients who received immunotherapy between 2010 and 2019 were screened, and 145 patients were included in the study per the inclusion criteria (**Table 1**). Of the sample, 59% (85/145) had pre-existing hypothyroidism. In the cohort of patients with pre-existing hypothyroidism (n=85), 15 had Hashimoto’s thyroiditis, 10 post-operative, 4 post-ablative, 3 medication-induced (sunitinib, amiodarone, pazopanib), while in 53 cases there was no documentation in the electronic medical records about the etiology of hypothyroidism. Forty-one percent (41%, 60/145) developed hypothyroidism [48 patients (80%) presented with primary hypothyroidism and 12 patients (20%) developed hypophysitis]. In the cohort of patients that developed hypophysitis with central hypothyroidism, 100% (12/12) had central adrenal insufficiency and were placed on either Hydrocortisone or Prednisone, while 17% (2/12, both male) also developed central hypogonadism and were placed on testosterone replacement therapy. Additionally, at least 30% of the patients with *de-novo* primary hypothyroidism experienced transient thyrotoxicosis during the first 12 weeks.

**Table 1:** Sample characteristics.

Characteristic	Pre-existing Hypothyroidism 59% (n = 85)	*De-novo* Hypothyroidism 41% (n = 60)		Total (n = 145)
		Hyphophysitis (n = 12)	Primary (n = 48)	
**Age (Years):**				
Median	71	63.5	65	68
*Range*	26-98	49-87	29-97	26-98
**Sex:**				
* Female*	48% (41)	17% (2)	65% (31)	51% (74)
* Male*	52% (44)	83% (10)	35% (17)	49% (71)
**Race:**				
*White Race*	77.6% (66)	83.3% (10)	77.1% (37)	77.9% (113)
*Other*	22.4% (19)	16.7% (2)	22.9% (11)	22.1% (32)
**Average weight (Kg):**				
* Prior to immunotherapy*	76.3	79.5	75.3	76.2
* Post immunotherapy*	75.9	79.4	75.4	76.0
**Doses of Immunotherapy:**				
*Mean*	7.13	6.67	13.5	9.19
*Range*	1-36	2-37	1-48	1-48
*Median*	4	4	7.5	4
**Types of Immunotherapy**				
**Combination PD-1 + CTLA4 inh**	18.8% (16)	33.3% (4)	29.2% (14)	23.5% (34)
* Pembrolizumab + Ipilimumab*	1.2% (1)	(0)	4.2% (2)	2.1% (3)
* Nivolumab + Ipilumumab*	17.6% (15)	33.3% (4)	25% (12)	21.4% (31)
**PD-1 inhibitors**	67.1% (57)	8.3% (1)	60.4% (29)	60% (87)
*Nivolumab*	35.3% (30)	8.3% (1)	33.3% (16)	32.4% (47)
*Pembrolizumab*	31.8% (27)	(0)	27.1% (13)	27.6% (40)
**CTLA4 inhibitors**	8.2% (7)	58.3% (7)	6.2% (3)	11.7% (17)
*Ipilumumab*	8.2% (7)	58.3% (7)	6.2% (3)	11.7% (17)
**PD-L1 inhibitors**	(0)	(0)	4.2% (2)	1.4% (2)
*Atezolizumab*	(0)	(0)	2.1% (1)	2.1% (1)
*Durvalumab*	(0)	(0)	2.1% (1)	2.1% (1)
Combination chemo + PD-1 inh*	5.9% (5)	(0)	(0)	3.4% (5)
**Number agents**				
*Monotherapy*	81.2% (69)	66.7% (8)	70.8% (34)	76.6% (111)
*Combination Therapy*	18.8% (16)	33.3% (4)	29.2% (14)	23.4% (34)
**Types of Cancer**				
*Melanoma*	29.4% (25)	83.3% (10)	41.7% (20)	37.9% (55)
*Kidney*	12.9% (11)	(0)	12.5% (6)	11.7% (17)
*Lung*	32.9% (28)	8.3% (1)	35.4% (17)	31.7% (46)
*Head and neck*	8.2% (7)	(0)	2.1% (1)	5.5% (8)
*Other*	16.5%(14)	8.3% (1)	8.3% (4)	13.1% (19)

Inh, inhibitors.

*Combination of Pembrolizumab + carboplatin + pemetrexed.

The studied group had a median age of 68 years, equal sex representation, and were predominantly of white race. No statistical difference was observed in the total sample’s average weight prior to and post immunotherapy (p=0.5). When compared to the pre-existing hypothyroidism group, the *de-novo* hypothyroidism group had lower average weights prior to and post immunotherapy. However, these differences did not prove to be statistically significant (p=0.5 and p=0.5, respectively).

The majority of patients had an initial diagnosis of melanoma or lung cancer and a smaller percentage was reported to have primary malignant involvement of the kidney, head/neck or other organs. There was wide variability in the number of doses of immunotherapy received, ranging from a minimum of 1 to a maximum of 48 with a median of 4. Interestingly, the *de-novo* primary hypothyroidism group had a higher median of 7 doses as compared to 4 in the pre-existing hypothyroidism and *de-novo* hypophysitis groups. Monotherapy was more prevalent than combination therapy, and the most common agents used were Nivolumab (32.4%) and Pembrolizumab (27.6%).

### Immunotherapy Effect on TSH Levels

**Figure 1:**
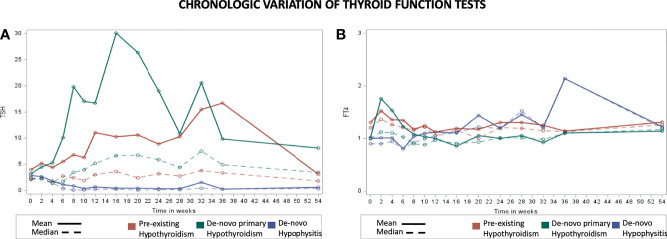
Chronologic display of thyroid function tests for the pre-existing hypothyroidism, *de-novo* primary hypothyroidism, and *de-novo* hypophysitis groups. X axis represents the number of weeks from initiation of immunotherapy and Y axis represents average TSH level (mIU/L) in part **(A)** and Free T4 level (ng/dL) in part **(B)**.

**Figure 2:**
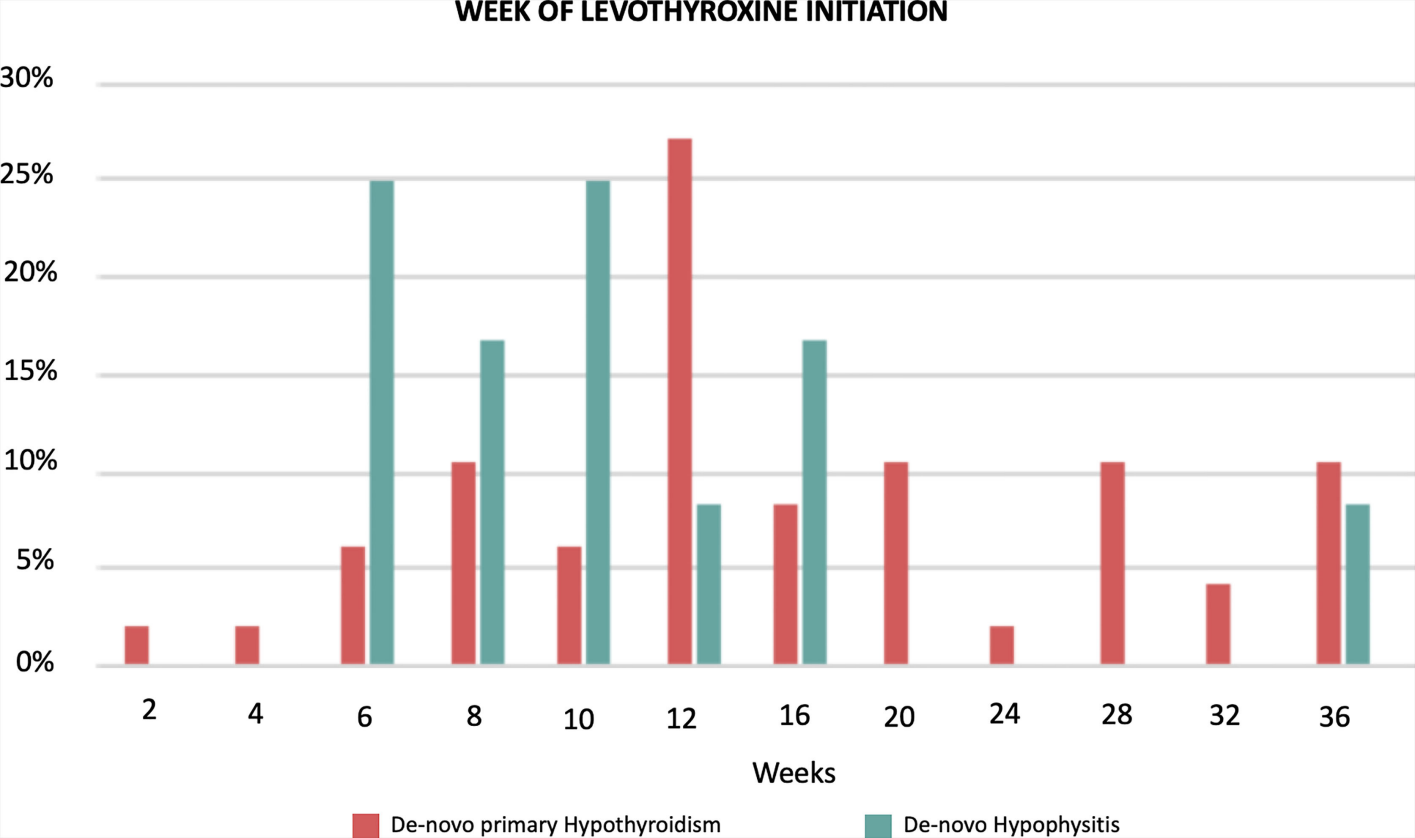
Timing of initiation of Levothyroxine in the *de-novo* primary hypothyroidism (n = 48) and hypophisitis (n = 12) groups. The data are presented as percentages with denominators being the total number of the *de-novo* primary hypothyroidism (n = 48) and hypophisitis (n = 12), respectively.

The participants from the pre-existing hypothyroidism group started experiencing TSH variations at week 6 with clinically significant TSH elevations between weeks 8 and 36 (highest mean TSH of 16.73 mIU/L at week 36) followed by progressive normalization of TSH levels towards week 54, with the appropriate adjustment of their levothyroxine dose (data presented below). Approximately 65% of the patients with pre-existing hypothyroidism remained with TSH levels <5 mIU/L during the 54 weeks. The *de-novo* primary hypothyroidism group experienced TSH variations starting at week 2 with the peak mean TSH being documented at week 16 (30.09 mIU/L), and progressive normalization afterwards (**Figure 1**; **Supplementary Table 1**). In general, higher TSH values were observed in the *de-novo* primary hypothyroidism group when compared with the pre-existing hypothyroidism group. Additionally, the *de-novo* hypophysitis group experienced persistently low mean TSH levels after week 8. The timing of initiation of levothyroxine for the *de-novo* primary hypothyroidism and hypophysitis groups is presented in **Figure 2**.

### Immunotherapy Effect on FT4 Levels

The pre-existing hypothyroidism group did not have clinically significant chronologic variations of mean FT4 levels (**Figure 1**; **Supplementary Table 2**). The *de-novo* primary hypothyroidism group experienced a progressive decline in mean FT4 levels starting at week 4 with the lowest level reported at week 16 (0.86 ng/dL), followed by progressive normalization to a mean level of 1.14 ng/dL by week 54. In the *de-novo* hypophysitis group, mean FT4 levels dropped to 0.8 ng/dL by week 4 and normalized to 1.22 ng/dL by week 54. Of note, the appropriateness of LT4 dosing in the *de-novo* hypophysitis group was assessed two-fold: 1) based on patients’ symptoms (lack of hypothyroid symptoms), and 2) LT4 dosing was titrated based on a FT4 goal in the upper half of normal range (close to the middle of the normal range). The timing of initiation of levothyroxine for the *de-novo* primary hypothyroidism and hypophysitis groups is presented in **Figure 2**.

### Trends of Levothyroxine Dosing After Initiation of Immunotherapy

**Figure 3:**
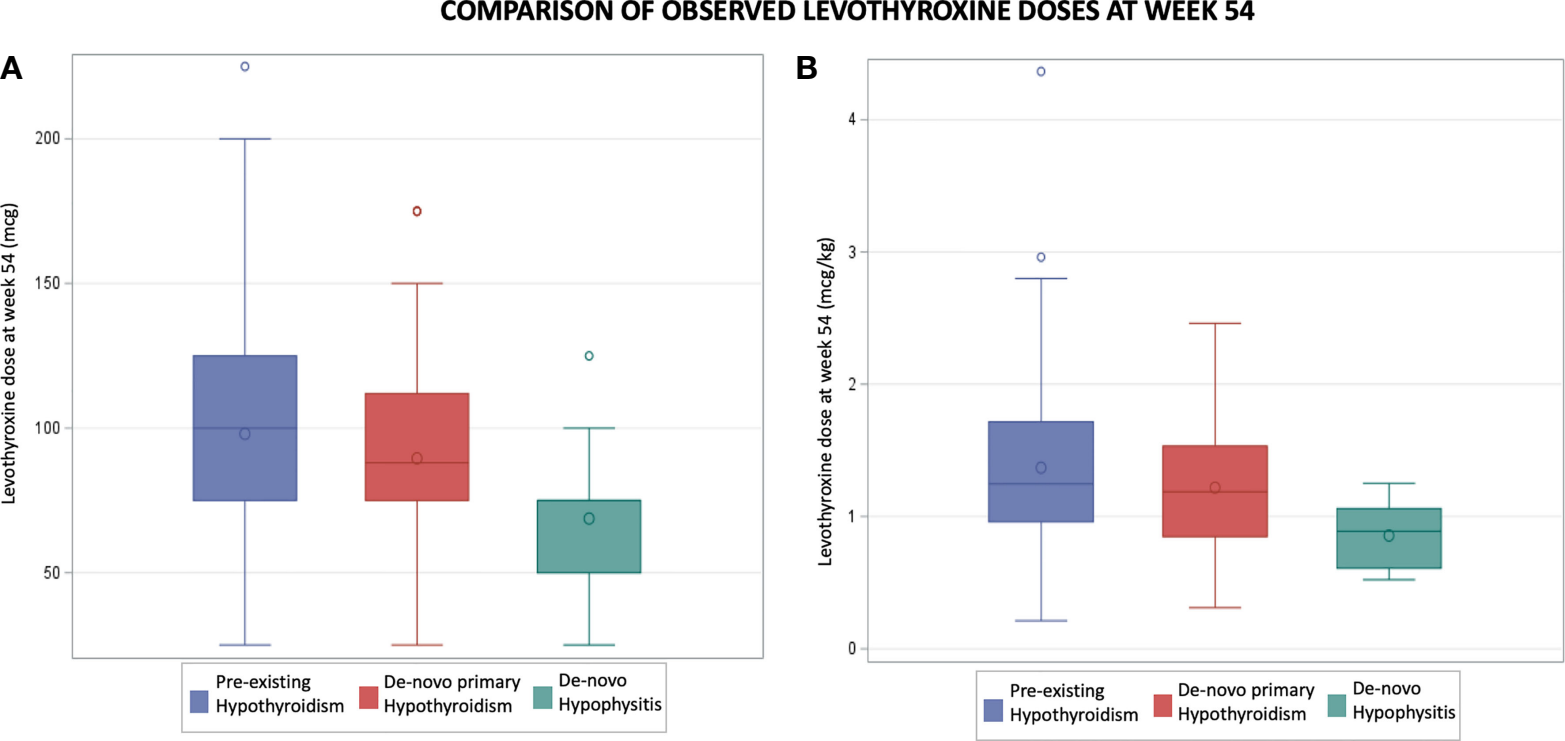
Comparison of observed levothyroxine doses at week 54. Part **(A)** compares mean levothyroxine doses in mcg. Part **(B)** compares mean levothyroxine doses in mcg per kg.

In the pre-existing hypothyroidism group, the final mean levothyroxine dose at week 54 was 98 mcg (SD 39) while the mean levothyroxine dose prior to ICI initiation was 92 mcg (SD 42). The mean difference of 6 mcg showed a trend towards statistical significance (p = 0.06). In this group there was no statistically significant difference in levothyroxine dosing at week 54 regardless of whether the patient had a TSH level >5 mIU/L at some point during the studied period. In the *de-novo* hypophysitis group, at week 54 the mean levothyroxine dose was 69 mcg (SD 26), which is statistically lower (p=0.003) than the pre-existing hypothyroidism (98 mcg, SD 39) and *de-novo* primary hypothyroidism (90 mcg, SD 38) groups (**Figure 3**).

Because of the statistically significant differences, and the need to translate those results in clinical practice, the observed mean levothyroxine dose was calculated based on body weight as mcg/kg. Patients with *de-novo* hypophysitis were found to have a statistically lower (p=0.009) observed mean dose in mcg/kg at week 54 (0.9 mcg/kg) as compared to the pre-existing and *de-novo* primary hypothyroidism groups (1.4 mcg/kg and 1.2 mcg/kg respectively).

When comparing the observed versus the calculated (weight based = 1.6 mcg/kg) levothyroxine doses at week 54 in the three groups, there were statistically significant lower observed means in all groups (**Table 2**). The biggest mean difference identified was in the *de-novo* hypophysitis group (mean difference: 63 mcg, p = 0.001); and the smallest, in the pre-existing hypothyroidism group (mean difference: 58 mcg, P<0.0001).

**Table 2:** Comparison of observed versus calculated (Weight based) Levothyroxine dose at week 54.

Group or subgroup	Observed Mean^1^ (SD)	Calculated (Weight based)^2^ Mean (SD)	Mean difference	p
*Pre-existing*	98.1 (39.5)	121.4 (33.4)	23.3	<0.0001
*De-novo primary hypothyroidism*	89.6 (38.5)	120.6 (27.7)	31.0	<0.0001
*De-novo hypophysitis*	68.8 (26.4)	127.0 (25.1)	58.3	<0.0001

^1.^Levothyroxine dose in mcg.

^2.^Calculated weight-based dose was obtained with the formula: 1.6mcg x kg.

**Figure 4:**
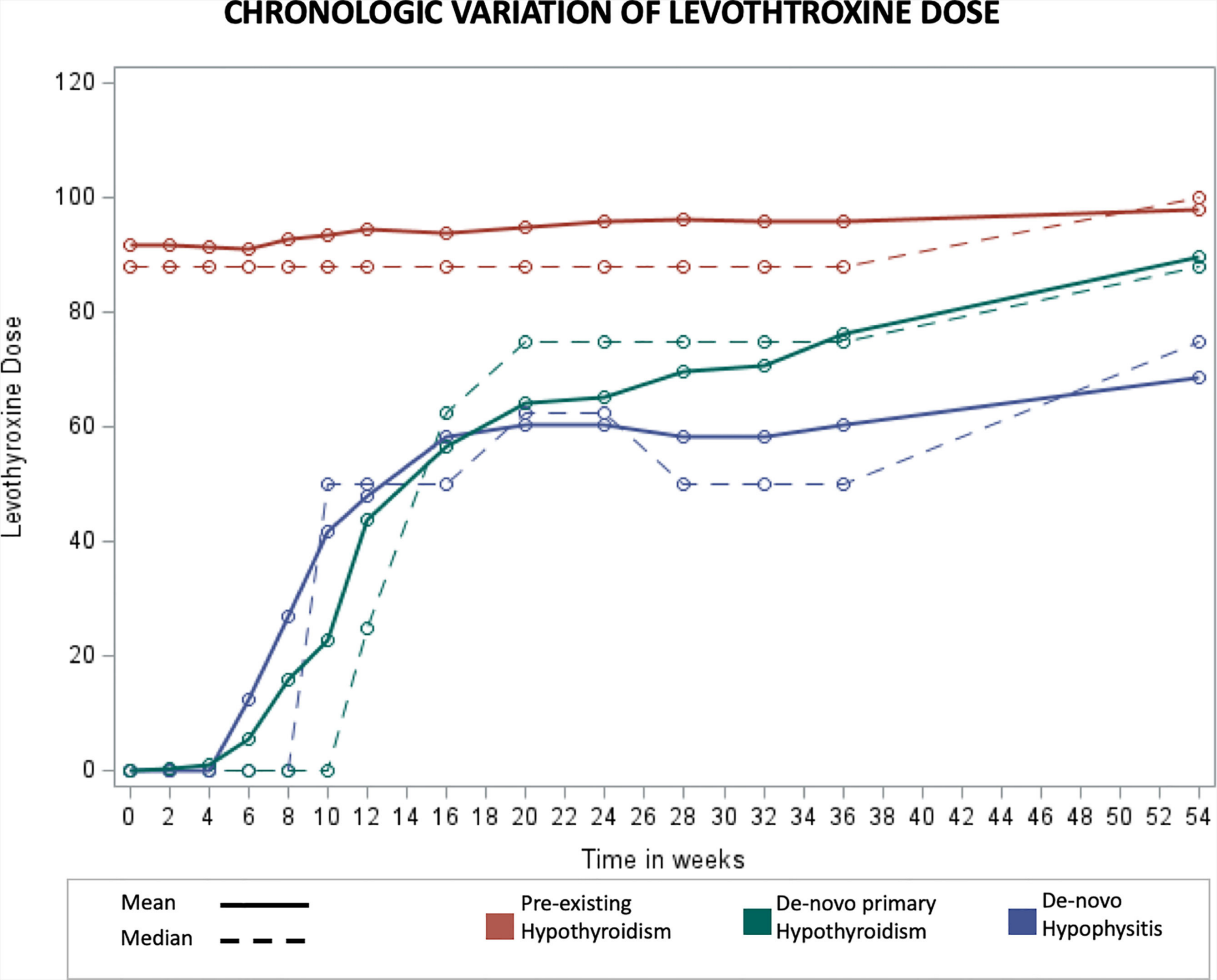
Chronologic display of mean Levothyroxine dose levels. X axis represents number of weeks from initiation of immunotherapy and Y axis represents mean Levothyroxine dose in mcg.

The chronologic variation of levothyroxine dosing was also evaluated (**Figure 4**). While in the pre-existing hypothyroidism group levothyroxine dosing remained relatively stable over time, in the *de-novo* hypophysitis group a sharp increase of 1.6 mcg per week was noted during the first 20 weeks, and a statistically lower weekly increase of 0.68 mcg/week was noted after week 20 (Mean difference -0.93 mcg, p <0.001).

## Discussion

The use of ICIs has revolutionized the treatment of cancer, and those agents are now the first line treatment for several malignancies. Although immunotherapies lack the side effect profile of traditional chemotherapies, they present with a rather unique constellation of adverse effects. Endocrinopathies are common immune-related side effects, and they can be very severe and must be carefully monitored during treatment with ICIs (22). Thyroid dysfunction is the most common among the immune-related endocrinopathies, and more data are needed to elucidate the clinical course as well as management (13).

In the current study, we present data on the immune-related thyroid dysfunction from an extensive cohort of patients treated in one of the largest healthcare systems in the Mid-Atlantic region of the United States. A total of 822 patients were included in the study, of whom 145 were found to have hypothyroidism and were further analyzed. We report an incidence of 7.2% (60/822) of *de-novo* hypothyroidism, of which 20% was secondary to hypophysitis while 80% was secondary to primary hypothyroidism. The incidence of *de-novo* hypothyroidism in the literature depends on the immunotherapy administered, and has been reported as low as 2% (23) and as high as 9.1% (24).

The mechanism by which immunotherapies cause thyroid dysfunction is still unclear. Orlov et al. reported that in 8 out of 10 patients with painless thyroiditis and hypothyroidism secondary to PD1 antibody treatment, anti-thyroglobulin and anti-thyroid peroxidase antibodies were detected, but no thyrotropin binding inhibitory immunoglobulins were found (25). The investigators hypothesized that polymorphic variants in the PD1 gene in some patients might predispose them to an increased risk of thyroid dysfunction. Unfortunately, because of the retrospective nature of the current study and the fact that measuring thyroid autoantibodies is not considered part of routine care of those patients, very few patients had thyroid autoantibodies values and no meaningful analysis could be performed. More prospective studies are needed in order to evaluate this potential pathophysiologic association.

The timeline of thyroid dysfunction was also studied. TSH variations were noted very early (2 weeks) after initiation of immunotherapy in the *de-novo* hypothyroidism group, while in the group with pre-existing hypothyroidism those changes were noted sometime later in the course of treatment (6 weeks). Those results support the findings from previous studies that immune related thyroid toxicity usually develops within the first 20 weeks of therapy, with symptoms of hypothyroidism first presenting after a median of 12 weeks in cases of primary hypothyroidism and 6 weeks in cases of transient thyrotoxicosis (11).

Despite the wide use of immunotherapy and the well-established thyroid side effect profile, there are no clear guidelines outlining the screening, surveillance and management of immune related thyroid toxicities. Initial recommendations from the American Society of Clinical Oncology (ASCO) include obtaining baseline thyroid function tests (TFTs) prior to initiation of immunotherapy and following TFTs every 4 to 6 weeks to screen for immune related thyroid toxicity or every 6-8 weeks to monitor treatment titration (26). Additionally, if immune related thyroid toxicity develops, ICIs can be continued unless there is evidence of severe or life-threatening symptoms and thyroid hormone therapy is the same as in non-immune related thyroid diseases. Based on the data generated from the current study, we suggest checking TFTs every 4 weeks until week 20, and decreasing frequency of TFTs to every 12 weeks after week 20. However, in patients that will be initiated on higher doses of immunotherapy or combination therapy, we suggest that the initiation of TFTs be at 2 weeks after initiation of treatment.

In the current study, we included and analyzed patients with pre-existing hypothyroidism that were started on immunotherapy. Prior studies have suggested that patients with pre-existing hypothyroidism should be closely monitored since they could develop severe hypothyroidism or thyrotoxicosis and could require adjustments in their levothyroxine dosing (16). In the current study, we demonstrate that patients with pre-existing hypothyroidism have much smaller variations in the TSH values after initiation of immunotherapy. Their levothyroxine dose was higher at week 54, but that difference did not reach statistical significance. Hence, we suggest continuation of the levothyroxine dose upon initiation of immunotherapy and serial TFT checks every 8-12 weeks thereafter.

Finally, in the current study the dosing of levothyroxine was studied extensively in both the pre-existing and the *de-novo* hypothyroidism groups. Per the ASCO initial recommendations (26), in patients with *de-novo* hypothyroidism thyroid hormone therapy should be initiated as in non-immune related thyroid diseases (1.6 mcg/kg/d or a starting dose of 50 mcg for elderly patients). However, in the current study we present data that shows that a lower dose of 1.2 mcg/kg levothyroxine might be sufficient to treat *de-novo* hypothyroidism. Importantly, by week 54, the overall sample and individual groups had achieved a biochemically euthyroid state as evidenced by median TSH and FT4 levels within the normal range. Similar findings were noted in a 2019 retrospective cohort analysis in which patients who developed primary hypothyroidism post ICI had an average levothyroxine maintenance dose of 1.29 mcg/kg (27). Based on our data, patients who develop primary hypothyroidism as a result of immunotherapy can be started at a dose of 1.2 mcg/kg/d with titration based on TFTs.

It has been observed that patients with secondary hypothyroidism may be euthyroid while taking weight-based doses of 1.5-1.6 mcg/kg (28) or 1.3-1.6 mcg/kg (29). The most pronounced difference from such weight-based estimates was noted in the *de-novo* hypophysitis group of patients in our study, such that these patients required 0.9 mcg/kg/d at week 54. Patients that develop immunotherapy-induced hypophysitis represent an older population with significant comorbidities. Hence the treating physicians are usually more conservative in titrating levothyroxine doses. Moreover, given the fact that the vast majority of this patient population also has other anterior pituitary hormonal deficits with overlapping symptoms, the treating physicians are cautious to avoid unnecessary over-replacement. For these reasons, we consider that in patients with hypophysitis secondary to immunotherapy it would be reasonable to start levothyroxine replacement therapy at approximately 50% of the recommended weight based dose (0.9 mcg/kg/day) and then titrate based on symptoms and FT4 goals to the upper half of the normal range.

This study has several limitations. Given the retrospective design without standardized protocols for TFTs monitoring and the use of different electronic medical records throughout the hospital system, it is possible that missing data on the baseline and biweekly TSH and FT4 levels could have led to the omission of patients with *de-novo* hypothyroidism and the underestimation of the frequency of transient thyrotoxicosis. Additionally, our results were not stratified per type of ICI given the size of the sample. Moreover, we could not collect TPO antibodies data as these were not clinically ordered for the vast majority of patients, and the very small number that was collected precluded any further meaningful analysis. The TPO antibody titer has been reported as a useful marker to predict both severity of thyroid toxicity and rates of occurrence. Despite these limitations, our results may inform current clinical practice and guide the design of future randomized prospective studies and inter hospital’s database collaborations to revise and strengthen the current findings and its derived suggestions.

In conclusion, the current retrospective review provides important insight into the management and treatment of hypothyroid patients receiving immunotherapy. A multidisciplinary approach is essential, and especially in patients with complex endocrinopathies the joint management by an oncologist and an endocrinologist would be extremely beneficial for the patients.

## Author Contributions

MK, JJ, and RG are responsible for the conception and design of the study. MK, DTT, and NF are responsible for data collection. MK, DTT, SD, and AB are responsible for data analysis and interpretation. DTT and AB are responsible for manuscript preparation. All the authors participated in the critical revision of the manuscript. All authors contributed to the article and approved the submitted version.

## Funding

Support for the current project was provided by a pilot grant from the Department of Medicine entitled ‘Effect of Immune Checkpoint Inhibitors on Thyroid Function: an Institutional Experience’ that was awarded to RG by the Department of Medicine at MedStar Georgetown University Hospital. The statistical analyses utilized in this publication were performed by the Biostatistics, Epidemiology, and Research Design core supported by the National Center for Advancing Translational Sciences of the National Institutes of Health under award number UL1TR001409. Data were presented in the form of an oral abstract entitled “Immunotherapy-associated Thyroid Disorders: Comparison of the Course of Pre-existing Hypothyroidism Compared with *De Novo* Hypothyroidism” at the 89th Annual American Thyroid Association Meeting (Chicago, IL – 11/2019).

## Author Disclaimer

The content is solely the responsibility of the authors and does not necessarily represent the official views of the National Institutes of Health.

## Conflict of Interest

The authors declare that the research was conducted in the absence of any commercial or financial relationships that could be construed as a potential conflict of interest.

## Publisher’s Note

All claims expressed in this article are solely those of the authors and do not necessarily represent those of their affiliated organizations, or those of the publisher, the editors and the reviewers. Any product that may be evaluated in this article, or claim that may be made by its manufacturer, is not guaranteed or endorsed by the publisher.
