# Supplementary material for: Immunotherapy-Associated Hypothyroidism: Comparison of the Pre-Existing With De-Novo Hypothyroidism
Source: Front Endocrinol (Lausanne). 2022 Mar 11;13:798253. doi: 10.3389/fendo.2022.798253 (PMC8962946; doi:10.3389/fendo.2022.798253)
Supplement: Supplementary file 1 [file DataSheet_1.docx]

**Supplementary Material**

**Supplementary Table 1: Chronologic variation of TSH after initiation of immunotherapy**

| **Week** | **Pre-existing Hypothyroidism**  Mean^1^ (SD) | **De-novo Hypothyroidism** | |
| --- | --- | --- | --- |
|  |  | **Primary**  Mean^1^ (SD) | **Hypophysitis**  Mean^1^ (SD) |
| ***0*** | 3.94 (6.03) | 3.16 (2.03) | 2.84 (1.75) |
| ***2*** | 5.22 (8.25) | 4.5 (13.33) | 2.70 (0.62) |
| ***4*** | 4.41 (9.32) | 5.25 (20.20) | 1.83 (1.62) |
| ***6*** | 5.54 (8.11) | 10.07 (24.27) | 0.14 (1.64) |
| ***8*** | 6.86 (10.73) | 19.81 (28.84) | 0.91 (1.49) |
| ***10*** | 6.29 (13.02) | 17.06 (23.71) | 0.39 (0.86) |
| ***12*** | 11.07 (21.18) | 16.71 (24.45) | 0.66 (1.35) |
| ***16*** | 10.27 (14.32) | 30.09 (46.31) | 0.48 (0.83) |
| ***20*** | 10.58 (15.70) | 26.37 (38.64) | 0.41 (0.75) |
| ***24*** | 8.88 (14.31) | 19.07 (27.46) | 0.34 (0.44) |
| ***28*** | 10.26 (17.70) | 10.83 (15.33) | 0.33 (0.43) |
| ***32*** | 15.48 (25.84) | 20.63 (35.01) | 0.47 (0.46) |
| ***36*** | 16.73 (29.42) | 9.85 (11.35) | 0.28 (0.38) |
| ***54*** | 3.07 (4.46) | 8.12 (12.41) | 0.61 (0.76) |

1. _TSH level in mIU/L._

**Supplementary Table 2: Chronologic variation of FT4 after initiation of immunotherapy**

| **Week** | **Pre-existing Hypothyroidism**  Mean^1^ (SD) | **De-novo Hypothyroidism** | |
| --- | --- | --- | --- |
|  |  | **Primary**  Mean^1^ (SD) | **Hypophysitis**  Mean^1^ (SD) |
| ***0*** | 1.30 (0.43) | 1.02 (0.14) | 1.00 (0.25) |
| ***2*** | 1.53 (0.63) | 1.76 (1.70) | 1.01 (0.23) |
| ***4*** | 1.35 (0.41) | 1.54 (1.37) | 1.01 (0.15) |
| ***6*** | 1.35 (0.58) | 1.23 (0.81) | 0.80 (1.04) |
| ***8*** | 1.17 (0.39) | 1.08 (0.70) | 1.04 (0.39) |
| ***10*** | 1.25 (0.49) | 1.04 (0.60) | 1.10 (0.35) |
| ***12*** | 1.12 (0.40) | 1.00 (0.38) | 1.12 (0.36) |
| ***16*** | 1.19 (0.47) | 0.86 (0.34) | 1.10 (0.32) |
| ***20*** | 1.18 (0.49) | 1.05 (0.80) | 1.44 (0.54) |
| ***24*** | 1.30 (0.53) | 1.00 (0.33) | 1.19 (0.14) |
| ***28*** | 1.30 (0.54) | 1.05 (0.29) | 1.45 (0.25) |
| ***32*** | 1.25 (0.56) | 0.92 (0.34) | 1.21 (0.30) |
| ***36*** | 1.14 (0.29) | 1.10 (0.39) | 2.14 (1.31) |
| ***54*** | 1.31 (0.35) | 1.14 (0.28) | 1.22 (0.25) |

1. _FT4 level in ng/dL._
